# Supplementary material for: Cryo-EM structures of the channelrhodopsin ChRmine in lipid nanodiscs
Source: Nat Commun. 2022 Aug 17;13:4842. doi: 10.1038/s41467-022-32441-7 (PMC9385719; doi:10.1038/s41467-022-32441-7)
Supplement: Supplementary file 1 — Supplementary Information [file 41467_2022_32441_MOESM1_ESM.pdf]

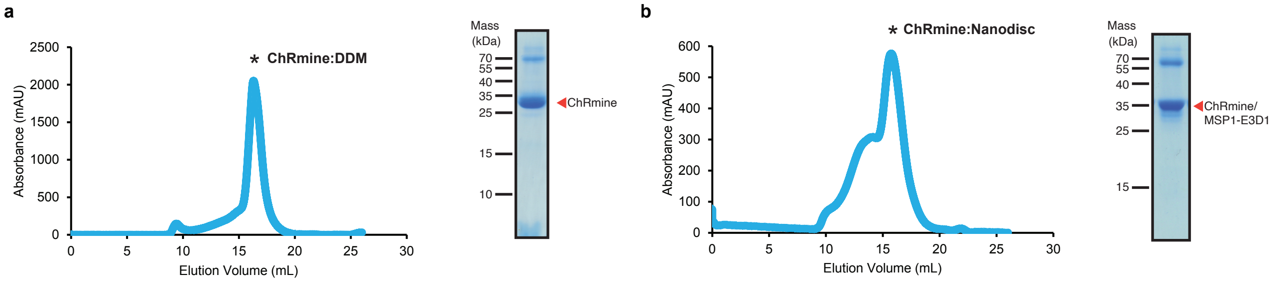

Supplementary Figure 1. ChRmine purification and nanodisc reconstitution.
(a,b) Chromatogram from a Superose 6 gel filtration of ChRmine **(a)** purified in DDM and **(b)** reconstituted into MSP1E3D1 nanodiscs. (right) Coomassie stained SDS–PAGE of pooled ChRmine-containing fractions indicated by stars above the chromatograms. Source data for **(a,b)** are provided as a Source Data file. Data are representative of n=3 purifications & reconstitutions.

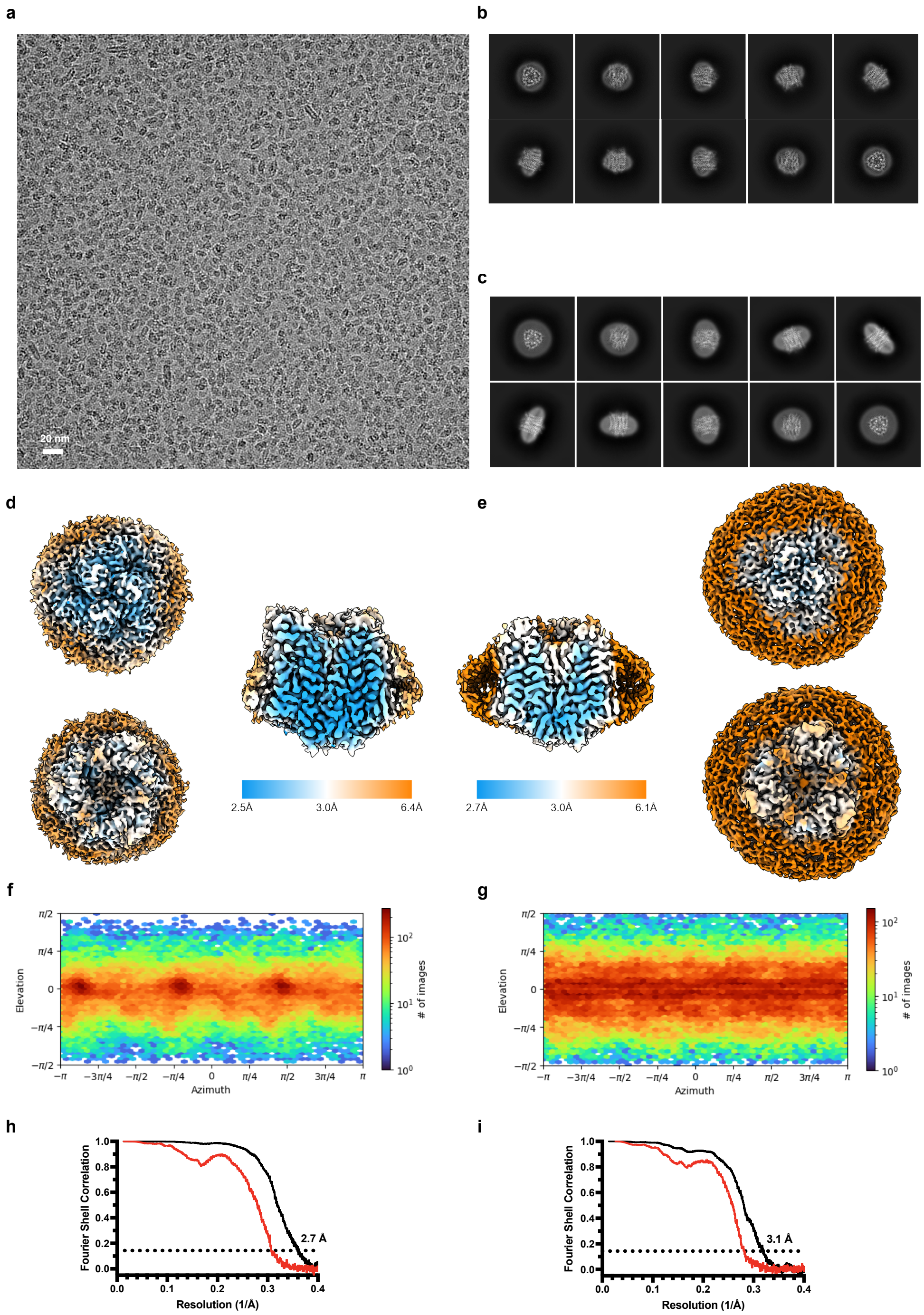

Supplementary Figure 2. Cryo-EM data and validation for retinal-bound ChRmine structures.
(a) A representative micrograph and **(b,c)** 2D class averages for ChRmine bound to all-trans retinal. **(b)** Small diameter and **(c)** large diameter nanodiscs were identified in the same dataset and processed separately. **(d,e)** local resolution drawn on final maps of **(d)** small diameter and **(e)** large diameter nanodiscs. **(f,g)** Angular distribution of particles in the final refinements of ChRmine in **(f)** small diameter and **(g)** large diameter nanodiscs. **(h,i)** Fourier Shell Correlation (FSC) between (black) masked and (red) unmasked half maps from refinement used for calculating resolution at FSC=0.143 from ChRmine in **(h)** small diameter and **(i)** large diameter nanodiscs. Displayed micrograph is representative of 10,900 movies collected (Table 1).

a

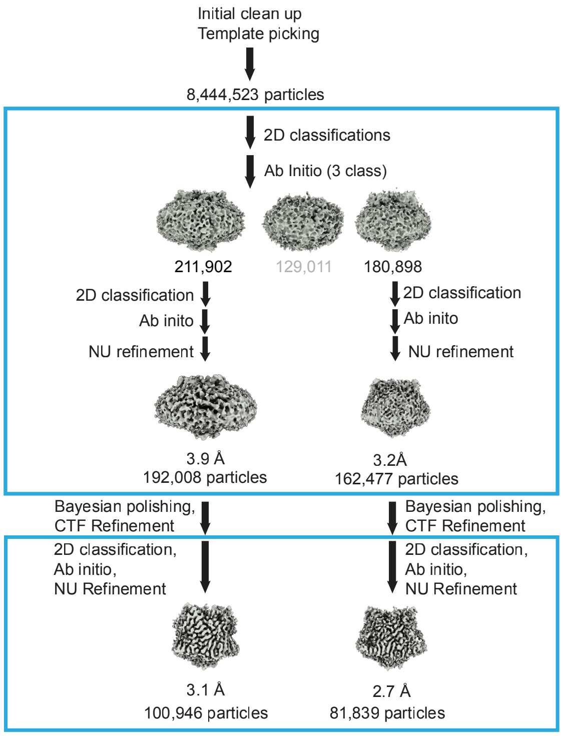

b

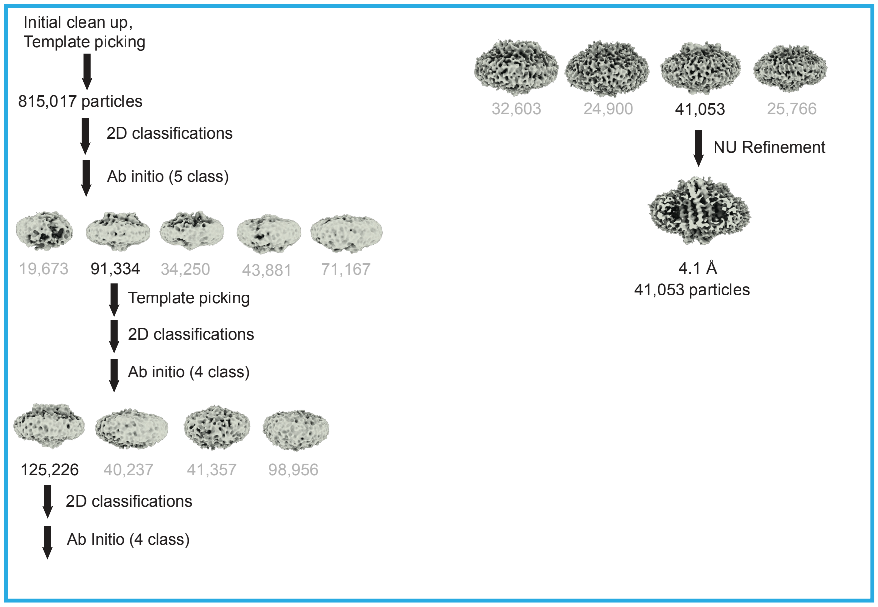

Supplementary Figure 3. Cryo-EM processing pipelines.
(a,b) Cryo-EM data processing steps for **(a)** retinal-bound ChRmine and **(b)** apo-ChRmine in lipid nanodiscs. See Methods for details**.**

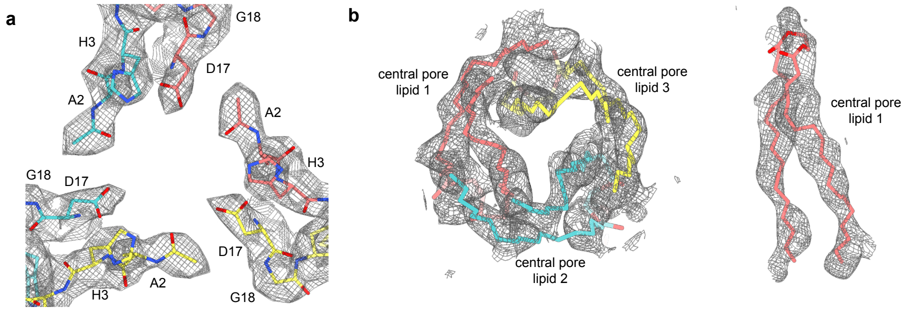

Supplementary Figure 4. Model and cryo-EM density for retinal-bound ChRmine.
(a) View from the extracellular side of the N-terminal region of ChRmine that forms the mouth of the central pore. The position of acetylated A2 is indicated. **(b)** View from the (left) intracellular side and (right) the membrane plane of central pore lipid in ChRmine. The model is shown within the final cryo-EM density.

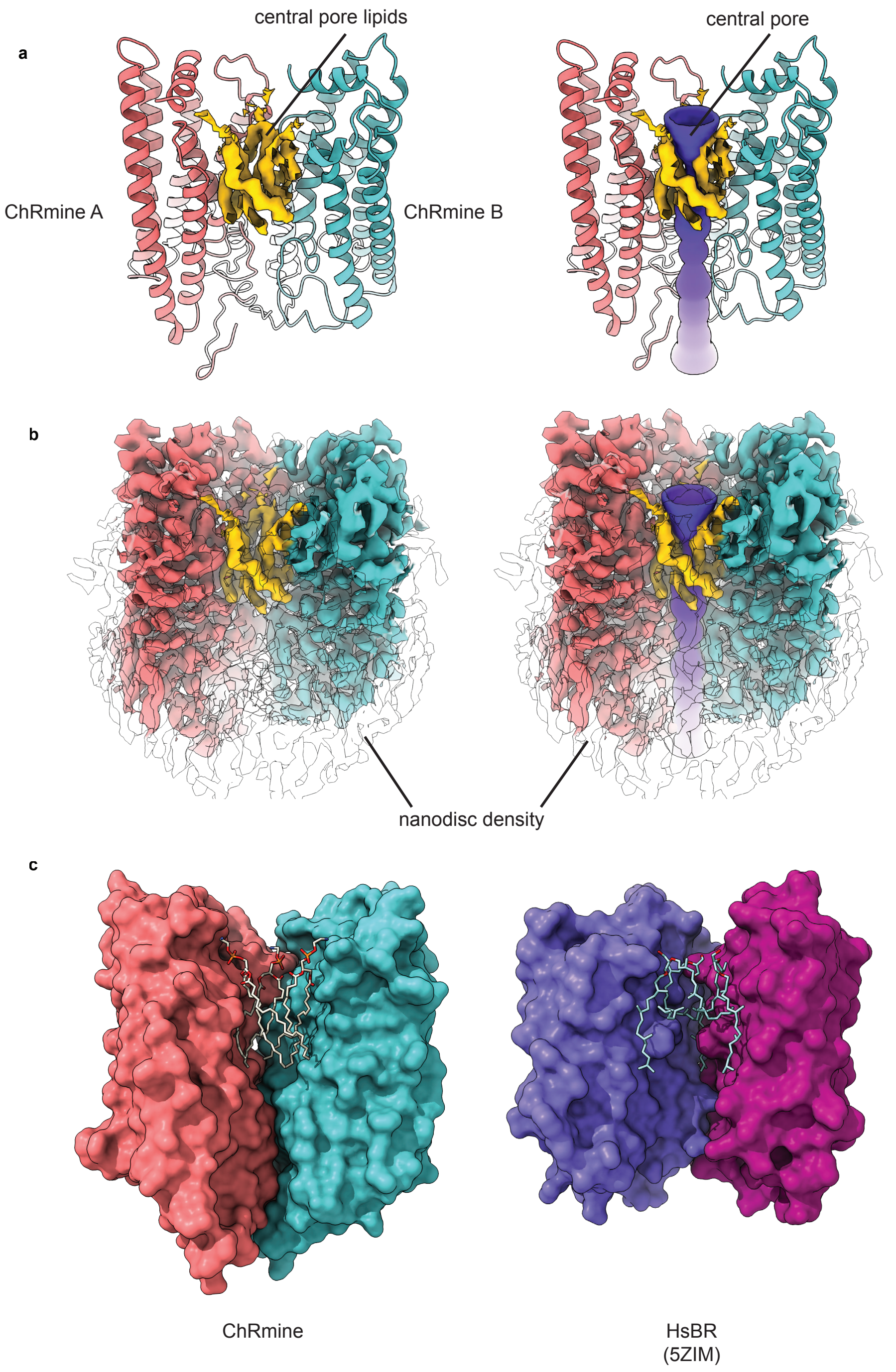

Supplementary Figure 5. Central pore lipids are occluded within the ChRmine trimer.
(a,left) Tilted view from the intracellular side of two ChRmine subunit models (red and teal) and pore lipid cryo-EM density (orange). **(a, right)** central pore profile is additionally shown and colored purple. **(b)** Same as **(a)** except with cryo-EM density for ChRmine subunits and lipid nanodisc shown. **(c)** Surface view of two subunits from (left) ChRmine and (right) HsBR with front subunit of each trimer removed. Bound lipids in the central pores are drawn as sticks. Bound lipids block ion conduction through the intersubunit pore and are occluded from bulk lipids by intracellular extensions of ChRmine that form a protein “fence” between the central pore and surrounding membrane.

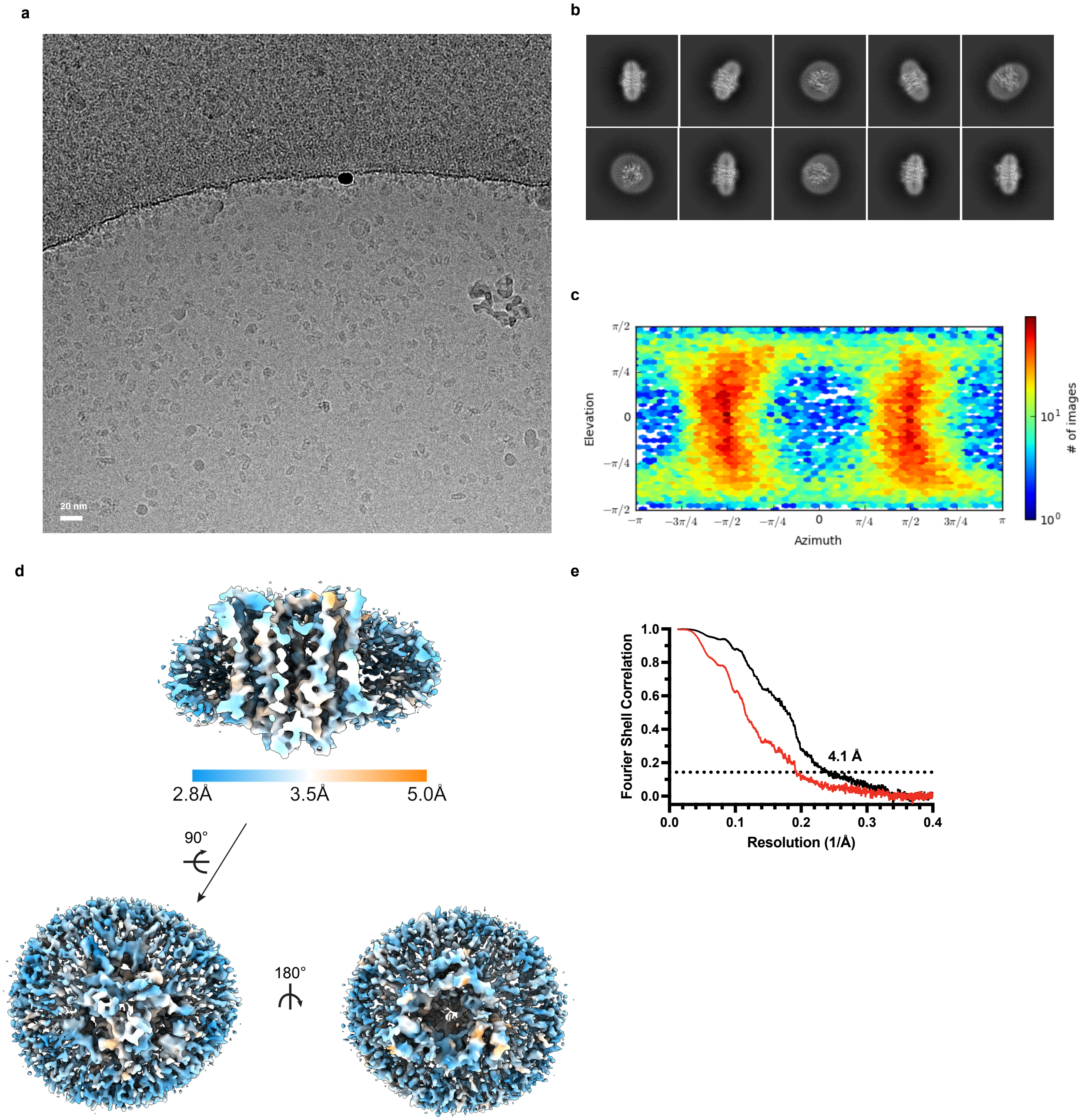

Supplementary Figure 6. Cryo-EM data and validation for apo ChRmine structures.
(a) A representative micrograph and **(b)** 2D class averages for apo-ChRmine in lipid nanodiscs. **(c)** Angular distribution of particles in the final refinement of apo-ChRmine. **(d)** Local resolution drawn on final apo-ChRmine map. **(e)** Fourier Shell Correlation (FSC) between (black) masked and (red) unmasked half maps from refinement used for calculating resolution at FSC=0.143. Displayed micrograph is representative of the 5,339 movies collected (Table 1).

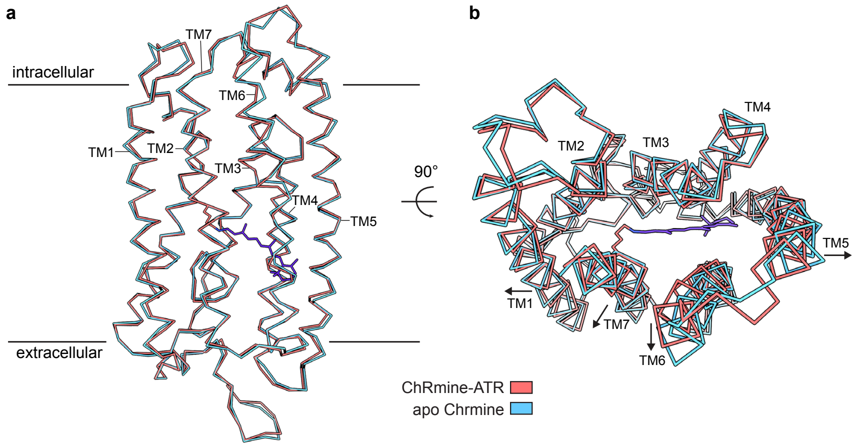

Supplementary Figure 7. Comparison of retinal-bound and apo ChRmine structures.
(a) Overlay of retinal-bound (red) and apo (blue) ChRmine from the membrane plane and **(b)** from the intracellular side. Positions and relative movements of transmembrane helices are indicated.
